# Rapid and Scalable Production of Functional SARS-CoV-2 Virus-like Particles (VLPs) by a Stable HEK293 Cell Pool

**DOI:** 10.3390/vaccines12060561

**Published:** 2024-05-21

**Authors:** Sitthiphol Puarattana-aroonkorn, Kannan Tharakaraman, Disapan Suriyawipada, Mathuros Ruchirawat, Mayuree Fuangthong, Ram Sasisekharan, Charlermchai Artpradit

**Affiliations:** 1 Program in Chemical Sciences, Chulabhorn Graduate Institute, Bangkok 10210, Thailand; sitthiphol@cgi.ac.th; 2 Koch Institute for Integrative Cancer Research, Massachusetts Institute of Technology, Cambridge, MA 02139, USA; 3 Translational Research Unit, Chulabhorn Research Institute, Bangkok 10210, Thailandmayuree@cri.or.th (M.F.); 4 Center of Excellence on Environmental Health and Toxicology (EHT), OPS, MHESI, Bangkok 10400, Thailand; 5 Program in Applied Biological Sciences, Chulabhorn Graduate Institute, Bangkok 10210, Thailand; 6 Department of Biological Engineering, Massachusetts Institute of Technology, Cambridge, MA 02139, USA

**Keywords:** virus-like particles (VLPs), stable cell pool, severe acute respiratory syndrome coronavirus 2 (SARS-CoV-2), vaccine, human embryonic kidney 293 (HEK293), coronavirus disease 2019 (COVID-19)

## Abstract

At times of pandemics, such as the severe acute respiratory syndrome coronavirus 2 (SARS-CoV-2) infection, the situation demands rapid development and production timelines of safe and effective vaccines for delivering life-saving medications quickly to patients. Typical biologics production relies on using the lengthy and arduous approach of stable single-cell clones. Here, we used an alternative approach, a stable cell pool that takes only weeks to generate compared to a stable single-cell clone that needs several months to complete. We employed the membrane, envelope, and highly immunogenic spike proteins of SARS-CoV-2 to produce virus-like particles (VLPs) using the HEK293-F cell line as a host system with an economical transfection reagent. The cell pool showed the stability of protein expression for more than one month. We demonstrated that the production of SARS-CoV-2 VLPs using this cell pool was scalable up to a stirred-tank 2 L bioreactor in fed-batch mode. The purified VLPs were properly assembled, and their size was consistent with the authentic virus. Our particles were functional as they specifically entered the cell that naturally expresses ACE-2. Notably, this work reports a practical and cost-effective manufacturing platform for scalable SARS-CoV-2 VLPs production and chromatographic purification.

## 1. Introduction

In the face of infectious disease outbreaks, bringing forward vaccines and therapeutics with the fastest timeline from discovery to clinical testing to ease burdens on healthcare systems and global socioeconomic issues is imperative. With significant time constraints during the pandemic of coronavirus disease 2019 (COVID-19), the pharmaceutical industry has been under immense pressure to deliver such preventive measures. Tremendous efforts toward biologics development led to a shortened production timeline to bring molecules to the investigational new drug (IND) application for human clinical trials [1,2]. Conventionally, material from a single clonal cell line is used for clinical development and manufacturing to ensure product quality. With recent improvements, the non-clonal approach eliminates the laborious and time-consuming steps of single-cell-derived clone screening [3]. This approach provides a potent strategy to reduce the time involved in the cell-line development process. This is beneficial during infectious disease outbreaks, where the rapid development of vaccines and therapeutics is essential due to time constraints. The accelerated path relies on non-clonal derived materials to enable earlier toxicology studies and phase I clinical trials [4,5,6]. This strategy can save almost four months compared to a single clone to generate the material [7]. One potential risk of using a cell pool approach is the likelihood of generating heterogeneous products, as distinct expression patterns exist among cells originating from a clonal cell line [8,9]. However, multiple studies indicate sufficient quality and comparable material when a stable pool is utilized for monoclonal antibody production [3,10,11]. The success of antibody development for COVID-19 has warranted adopting a non-clonal concept for biologics production [12].

As we have seen in the case of the COVID-19 pandemic, various vaccine platforms are being used to obtain vaccines with good safety profiles and high efficiency. Developing a vaccine candidate requires careful consideration of the interactions between the vaccine antigen and the host. While traditional approaches like inactivated viral particles and subunit vaccines can stimulate CD4+ T_h_ cells (T-helper cells) and humoral immunity, their effectiveness may be limited due to alterations in antigen structure during production. This hinders optimal engagement with the host’s immune repertoire. Furthermore, these approaches are poor inducers of cytotoxic CD8+ T cells, which are crucial for an effective COVID-19 vaccine [13,14,15]. Subunit vaccines also lack the repetitive antigenic structure on their surface, which is essential for efficient recognition by immune cells. More advanced platforms, such as recombinant viral vectors and mRNA vaccines, have emerged as successful approaches to combat COVID-19. However, each approach poses specific challenges in terms of host immune responses [16,17,18,19]. Virus-like particles (VLPs) have an advantage over other vaccines because they mimic the natural viral structure and present antigens in their native conformation. This makes it easier for our immune system to recognize them effectively. VLPs are also able to interact with our immune system’s dendritic cells through the recognition of viral pathogen-associated molecular patterns (PAMPs) using pattern recognition receptors (PRRs), such as toll-like receptors (TLRs) [20]. This interaction leads to a strong immune response, including both humoral and cell-mediated immunity [21]. Additionally, VLPs have a high safety profile because they lack genetic material and cannot replicate. Due to their ability to elicit strong immune responses through precise molecular mimicry and interaction with the human host’s immune system, VLPs hold immense promise for future vaccine development.

SARS-CoV-2 VLPs production has been extensively reported [21,22]. The particles can be classified into two subtypes: heterotypic (chimeric) VLPs, where the spike protein (S) is displayed on heterologous VLP scaffolds, and homotypic VLPs that are based on SARS-CoV-2 structural proteins. The chimeric vaccine approach has been investigated as a potential strategy for combating various viruses, such as using multiple glycoproteins of Epstein-Barr virus (EBV) as the surface antigen and incorporating the structural protein of Zika virus as the antigen on another viral scaffold [23,24]. Six VLPs-based vaccines of SARS-CoV-2 are currently in clinical studies, and only one candidate co-expresses the spike (S), envelope (E), membrane (M) proteins, and nucleocapsid (N) of the virus [22]. Not only is the S protein capable of immune induction but multiple studies have shown that other structural proteins are immunogenic [25,26,27,28]. This makes homotypic SARS-CoV-2 VLPs a more attractive target than the chimeric type. Furthermore, it has been observed that the spike protein exhibits greater immunogenicity when present in a stabilized prefusion conformation, as compared to the wild-type spike protein [29]. This conformational alteration is achieved by introducing two proline substitutions (2P) in the S2 subunit, enhancing the recombinant protein expression [29,30]. In addition to the 2P substitution, mutating the furin cleavage site to prevent the S1/S2 cleavage improves protection in animal studies [31,32]. It is of particular interest to assess the potential effect of these mutations on VLP assembly and the spike incorporation when utilizing the S mutants.

Various expression systems have been implemented to produce homotypic SARS-CoV-2 VLPs based on transient transfection and a stable cell pool [33,34,35]. Mammalian cell lines are particularly preferred over insect and bacterial cells due to their ability to carry out more complex glycosylation. Therefore, interest has been drawn to the use of a stable human embryonic kidney 293 (HEK293) cell pool for biotherapeutics and vaccine production. HEK293 can be grown in high-density suspension with high yield and possesses human post-translational modifications (PTMs) [36]. Targeted integration methods to create a stable cell pool, such as recombinase-mediated cassette exchange and transposase-mediated integration, have become a recent trend [37,38,39]. This type of stable pool generation may bestow good transgene expression since optimal loci for integration can be selected. However, this method is less accessible due to the proprietary recombinases and genome loci validation needed for the exchange [40]. The simple transfection of plasmid vectors bearing genes of interest with an antibiotic selection to create a stable cell pool is more suitable for early-stage development. The stable cell pool for expressing recombinant proteins can be established quickly with readily available and cheap reagents such as polyethylenimine (PEI) that provides a random DNA integration pool approach. PEI is a molecule that can bind to DNA via electrostatic interaction and serves as a carrier. The positively charged PEI–nucleic acid complex can interact with the negatively charged cell membrane and promote cellular uptake through endocytosis [41]. Many homotypic SARS-CoV-2 VLPs expressed in mammalian cells suffered from upscaling due to their expression setting and downstream processing [42,43,44,45,46]. A scalable VLPs production with a mammalian expression platform is vital in response to pandemics to rapidly deliver new vaccines toward IND-enabling toxicology study. The development of such a platform is critical to ensure that the VLPs can be swiftly produced in large quantities, efficiently, and cost-effectively. The implementation of the cell pool approach in producing VLPs has a significant advantage in proactively generating VLPs that target not only newly emerging SARS-CoV-2 variants but also other upcoming viral infectious diseases. It is crucial to respond rapidly to emerging diseases to control the spread of the virus and mitigate the burden of disease, especially in terms of host–virus interaction.

In this study, we established a quick and upscalable mammalian cell expression platform that can produce functional SARS-CoV-2 VLP. Spike proteins based on the variant BA 2.75 and E-M (envelope–membrane) were co-expressed in HEK293-F cells. Our expression design offers adaptability since S is encoded in a separate vector. We could simply add a spike from new variants to express the VLPs. Additional mutations were introduced to the spike protein to study its effect on VLPs’ assembly. We employed a simple transfection method with a widely used chemical agent, PEI, to create a stable cell pool. The particle expression was stable for over a month. Different feeds were studied to increase the expression yield. The structure of expressed particles was assessed using a transmission electron microscope (TEM). The size distribution of the purified VLPs was determined by dynamic light scattering (DLS). Finally, we showed that our SARS-CoV-2 VLPs were functional, as the transduction of the VLPs into the target cells was observed.

## 2. Materials and Methods

### 2.1. Cell Lines and Culture Media

Freestyle™ 293-F cells (Thermo Fisher Scientific: R79007, Waltham, MA, USA) were cultured in Freestyle™ 293 Expression Medium (Gibco, Paisley, UK). Freestyle™ 293 Expression Medium is an animal origin-free, protein-free, and chemically defined medium used for passaging, transfection, and VLPs production. The cells were passaged two to three times per week at a density of 0.3 × 10^6^ cells/mL and maintained at 37 °C, 125 rpm, and 8% CO_2_. Calu-3 cells (ATCC: HTBB-55; human lung adenocarcinoma cell line, Manassas, VA, USA) were cultured in Eagle’s minimum essential medium (EMEM) (ATCC, Manassas, VA, USA) supplemented with 10% fetal bovine serum (FBS) (ATCC, Manassas, VA, USA). A549 cells (ATCC: CCL-185; human lung carcinoma cell line, Manassas, VA, USA) were maintained in Dulbecco’s modified Eagle medium/Nutrient Mixture F-12 (DMEM/F-12) (Gibco, Grand Island, NY, USA) supplemented with 10% FBS. Both cultures were kept under 5% CO_2_ and 37 °C. All media were chemically defined and free of other animal components.

### 2.2. Genetic Constructs and Transfection

Genes encoding the SARS-CoV-2 spike of the BA 2.75 variant (GenBank: UTM82166.1) and SARS-CoV-2 E-M (GenBank: NC_045512) of the wild-type variant were codon optimized for expression in mammalian cells. The pcDNA3.1 (+) plasmid was used as an expression vector. Site-directed mutagenesis (SDM) was employed to generate mutations on the spike, i.e., prefusion stabilization mutations or 2P mutation (K983P and V984P) and furin cleavage site mutations (R680S and R682G). SDM reactions were conducted using a QuikChange Lightning Site-Directed Mutagenesis Kit (Agilent, Cedar Creek, TX, USA). Recombinant plasmids were then transfected into the HEK293-F cells. Briefly, the cells were seeded with an 80 mL culture volume at 0.5 × 10^6^ cells/mL overnight in a 250 mL flask. When the cells reached 1 × 10^6^ cells/mL, fresh Freestyle™ 293 Expression Medium was replenished. pcDNA3.1 (+)—E-M and pcDNA3.1 (+)—S were used to transfect the cells at the molar ratio of 1:2, having a total of 1.25 µg of DNA per 1 mL culture and using polyethyleneimine (PEI-MAX, PolyScience, Warrington, PA, USA) at a mass ratio of DNA:PEI of 1:3.5. Three different constructs of the spike containing plasmid (no mutation, 2P mutation, and 2P + furin cleavage site mutation) were separately co-transfected with the envelope–membrane-containing plasmid.

### 2.3. Stable Cell Pool Selection and Stable Expression of SARS-CoV-2 VLPs

Various concentrations of geneticin (Gibco, Grand Island, NY, USA) were tested in preliminary experiments conducted with HEK293-F cells. The addition of 100 µg/mL of geneticin concentration was selected. The antibiotic was added at 48 h after transfection. Medium with geneticin was replenished every 3–4 days. The frozen cell stocks were generated after the selection process. The assembly of SARS-CoV-2 VLPs was assessed by discontinuous sucrose gradient ultracentrifugation. Twenty-five milliliters of supernatant from transfected cultures was clarified by centrifugation at 4000× *g* for 10 min and subjected to 0.2 µm filtration. The supernatant was overlaid onto a 20–60% sucrose gradient. Ultracentrifugation was operated at 28,000 rpm (133,900× *g*) using Beckman SW32Ti rotors for 2.5 h at 4 °C. Fractions of the gradient were collected (1 mL/fraction). The presence of VLPs was identified using a Western blot. The expression level of the spike and membrane proteins of the selected construct (2P mutation) was checked at every passage to evaluate the stability of SARS-CoV-2 VLPs production. In brief, 1 mL of culture supernatant was collected and centrifuged at 200× *g* for 5 min. The protein expression in the supernatant was analyzed using Western blot analysis.

### 2.4. Western Blot

Samples were prepared by resuspending with loading dye containing sodium dodecyl sulfate (SDS) and β-mercaptoethanol (Calbiochem, Shanghai, China) and boiled for 5 min. Samples were resolved using 10% or 12% SDS-polyacrylamide gel electrophoresis (SDS-PAGE). Proteins were electrotransfered onto a nitrocellulose membrane. The membranes were blocked with Tris-buffered saline containing 0.05% tween-20 (Merck, Alsace, France) and 5% bovine serum albumin (BSA) (Sigma-Aldrich, Saint Louis, MO, USA) for an hour, followed by overnight incubation with rabbit anti-SARS-CoV-2 S antibody (1:2000, ab281310, Abcam, Hangzhou, China), rabbit anti-SARS-CoV-2 M antibody (1:2000, NBP3-07058, Novus Biologicals, Centennial, CO, USA) or rabbit anti-SARS-CoV-2 E antibody (1:1000, NBP3-070560, Novus Biologicals, Centennial, CO, USA) and then an hour incubation with a secondary antibody, anti-rabbit IgG conjugated with HRP (1:10,000, ab6721, Abcam, Hangzhou, China). The membranes were developed using Amersham ECL Prime Western Blotting Detection Reagent (Cytiva, Amersham, UK). The ImageQuant™ LAS 4000 biomolecular imager and ImageQuant™ TL software version 10.2 (GE, Uppsala, Sweden) were used for image acquisition and image processing, respectively. Digitization images (red, protein molecular weight ladder) were exposed simultaneously with chemiluminescence images (green, antibody-probed proteins).

### 2.5. Feed Screening for a Fed-Batch Experiment on a Small-Scale Production

The HEK293-F cell pool with the 2P mutation was subjected to a feed screening study with three different supplements, CD EfficientFeed™ C AGT (Gibco, Grand Island, NY, USA), Resurge™ CD3, and Resurge™ CD4 (Gibco, Miami, FL, USA). Fifteen percent of the final volume of CD EfficientFeed™ C AGT was added on Day 3, while Resurge™ CD3 and CD4 were supplemented on Day 0 at 3 g/L. Cell growth and viability were monitored daily. Supernatants were collected daily. The spike protein was quantified using immunoblot analysis.

### 2.6. Dot Immunoblot 

The BA2.75 spike standard (R&D systems, Rochester, MN, USA) was diluted at preferred concentrations. Standards and samples were boiled at 95 °C. Two microliters of standards and samples were dotted onto a nitrocellulose membrane. Spike proteins were detected as described in the Western blot section. Standard curves for spike protein were constructed based on the intensity exposure of multiple standard concentrations. The spike concentration from each condition was determined by extrapolation from the standard curve.

### 2.7. Production and Purification of SARS-CoV-2 VLPs in a Stirred-Tank Bioreactor

Upscalability for SARS-CoV-2 VLPs production was assessed in a 2 L stirred-tank bioreactor (Biostat^®^ B, Sartorius, Goettingen, Germany). The cells were seeded at a density of 0.3 × 10^6^ cells/mL in a final volume of 1.5 L in Freestyle™ 293 Expression Medium, supplemented with 15% CD EfficientFeed™ C AGT™ nutrient supplement on Day 3 without antibiotic selection. Dissolved oxygen (DO) was maintained at 40% with a constant airflow head space at 10 mL/min. Sparged pure air and pure oxygen were automatically adjusted by the BIOPAT^®^ DCU local controller software (https://www.sartorius.com/en/products/process-analytical-technology/process-control-automation/biopat-dcu, accessed on 9 May 2024, Sartorius, Goettingen, Germany). The pH setpoint was at 7.1, temperature at 37 °C, and stirring speed at 100 rpm. The pH was regulated only by CO_2_ addition. Two experiments of a stirred-tank bioreactor culture for SARS-CoV-2 VLPs production were performed independently. The supernatant was collected daily to observe cell growth using the Countess 3 Automated Cell Counters (Thermo Fischer Scientific, Carlsbad, CA, USA) and to analyze metabolites using the Biomedical BioProfile (NOVA, Waltham, MA, USA). The culture was harvested seven days post-seeding. The supernatant was collected by centrifugation at 4000× *g* for 10 min and clarified with 0.2 µm filtration. The sample was then purified using a chromatographic approach. The SARS-CoV-2 VLPs were purified using CaptoCore 700 (92 mL column) with an ÄKTA pure™ chromatography system (Cytiva, Uppsala, Sweden) in flowthrough mode with a running buffer containing 20 mM Tris and 150 mM NaCl, at pH 7.0. Collected supernatants were loaded into the column at 11 mL/min. The flowthrough samples were concentrated and buffer-exchanged to the running buffer using Amicon Ultra Centrifugation Filters 100 kDa (Merck, Darmstadt, Germany). Purified VLPs were used for further characterization.

### 2.8. Transmission Electron Microscopy (TEM)

Purified VLPs were adsorbed onto copper grids with a Formvar/Carbon support film (VWR, Radnor, PA, USA). After drying, the samples were stained with 1% uranyl acetate (Electron Microscopy Sciences, Hatfield, PA, USA). Grids were then washed with water and air-dried. The SARS-CoV-2 VLPs were visualized using HT7700 transmission electron microscopy (Hitachi, Tokyo, Japan).

### 2.9. Particle Size Analysis by Dynamic Light Scattering (DLS)

The hydrodynamic particle size of SARS-CoV-2 VLPs was analyzed using a particle size analyzer, DynaPro^®^ NanoStar^®^ (Wyatt Technology, Santa Barbara, CA, USA). The purified samples were measured in native liquid condition with a disposable cuvette. The samples were maintained at room temperature. Measurements were collected with 5 s of acquisition time for 10 acquisitions. The final hydrodynamic diameter and distribution were obtained using DYNALS software version 2.9.1 (Wyatt Technology, Santa Barbara, CA, USA).

### 2.10. SARS-CoV-2 VLPs Receptor Binding Assay

Calu-3 and A549 cells were plated in 4-well chamber slides at a density of 4 × 10^4^ cells/mL. At 24 h post-seeding, cells were washed with phosphate-buffered saline (PBS) and treated with 300 ng of purified VLPs at 37 °C for 2 h under 5% CO_2_. Subsequently, the cells were fixed with 4% formaldehyde (Invitrogen, Grand Island, NY, USA) and permeabilized with 0.2% Triton-X 100 (PanReac AppliChem, Barcelona, Spain). Rabbit anti-SARS-CoV-2 M antibody (1:200, NBP3-07058, Novus Biologicals, Centennial, CO, USA) and rabbit polyclonal anti-SARS-CoV-2 S antibody (1:100, ab272504, Abcam, Hangzhou, China) were used to detect SARS-CoV-2 VLPs in the cells, while rabbit anti-ACE-2 (1:250, sc-390851, Santa Cruz Biotechnology, Dallas, TX, USA) was applied to probe ACE-2. Anti-rabbit IgG conjugated with Alexa Fluor^®^ 647 (1:250, ab150079, Abcam, Hangzhou, China) and anti-mouse IgG conjugated with Alexa Fluor^®^ 488 (1:250, AB_2338840, Jackson ImmunoResearch, Philadelphia, PA, USA) were used as a secondary antibody for anti-SARS-CoV-2 M or anti-SARS-CoV-2 S and anti-ACE-2 detection, respectively. Cell nuclei were stained with Hoechst 33342 (2 µg/mL) (Molecular Probes, Eugene, OR, USA). The chamber slides were visualized using an FV3000 Confocal Laser Scanning Microscope (Olympus, Tokyo, Japan) equipped with FV31S-SW software version 2.6 for image acquisition and processing. ImageJ version 1.53t was used for image adjustment [47].

## 3. Results

### 3.1. Generation of a Stable HEK293-F Cell Pool Expressing SARS-CoV-2 VLPs

**Figure 1:**
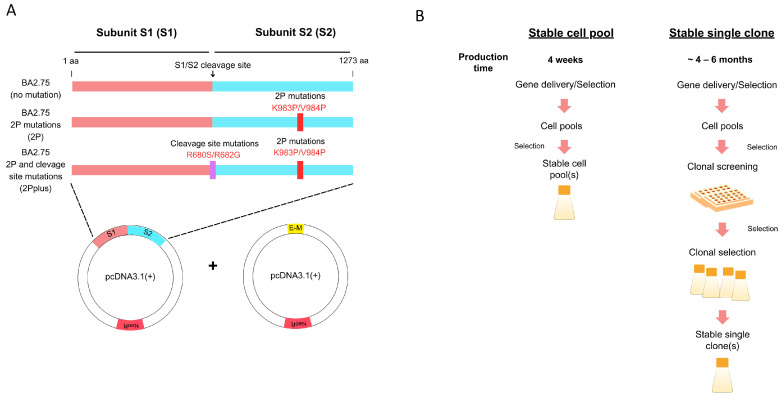
Design of SARS-CoV-2 VLPs production. (**A**) Genetic constructs of the spike (S) and envelope–membrane (E-M) genes. The spike from the BA2.75 variant was used, while the envelope and membrane were of the original strain of SARS-CoV-2. The specified mutations were created from site-directed mutagenesis reactions. (**B**) Workflow and timeline comparison between the stable cell pool and stable single-cell clone strategies to produce recombinant proteins.

To create a stable HEK293-F cell pool, the cells were co-transfected with plasmids encoding the spike protein (monocistronic vector) and envelope–membrane protein (bicistronic vector). Three designs of the spike gene of the BA 2.75 strain were chosen: no mutation, K983P and V984P mutations (2P), and 2P mutations plus furin cleavage site mutations at R680S and R682G (2Pplus) (Figure 1A). We investigated whether mutations on the spike protein impact VLP assembly. Therefore, three different HEK293-F cell pools were generated separately with three types of spike mutations by transfection. The transfection was performed with PEI. Following the antibiotic addition, cell viability declined in all three transfection reactions. After a week, the cell viability dropped to 50%. The cells slowly recovered after two weeks, with more than 90% viability. Cells were further maintained under the selection for another week to fully recover their doubling time before generating the cell stocks. The generation of stable cell pools could be completed in under a month. Figure 1B shows that the development of a stable cell pool can be achieved in a shorter timeline when compared to the typical stable single clone development. The scaling-up process of a stable cell pool expressing SARS-CoV-2 VLPs was further evaluated.

**Figure 2:**
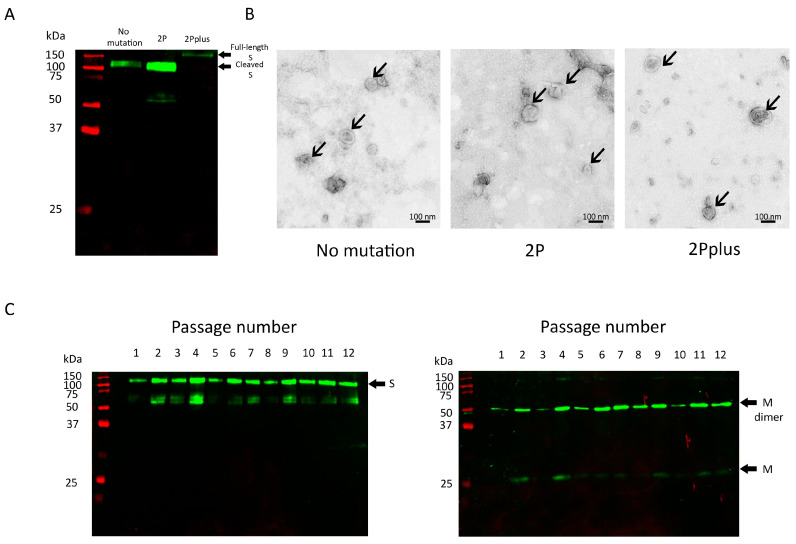
SARS-CoV-2 VLPs production from different spike constructs. (**A**) Analysis of spike incorporation using a Western blot probed with anti-S antibody. (**B**) TEM images of sucrose gradient centrifugation-purified SARS-CoV-2 VLPs produced from stable HEK293-F cell pools. The arrows indicate VLPs. (**C**) Consistency of expression of spike and membrane proteins from the 2P mutation stable cell pool. Lane 1–12: culture supernatant collected from different cell passages. Cell passages were performed every 3–4 days.

For protein production, the stable cell pools were cultured without the antibiotic. Culture supernatants were harvested after seven days of culture. The proteins from three spike constructs were subjected to sucrose gradient ultracentrifugation and TEM. VLP formation was detected using a Western blot with an anti-spike protein. The spike protein derived from all three designs was predominantly detected in Fractions 4–8 of the sucrose gradient solution. The spike protein from the 2P design showed higher spike incorporation than the no mutation design and 2Pplus design, as the expression from the 2P design was relatively higher than the other two constructs (Figure 2A). The spike protein from the constructs without mutations in furin cleavage (no mutation and the 2P mutation design) was detected at ~100 kDa. A study from Boson et al. has reported that when the SARS-CoV-2 spike is expressed in HEK293-F cells, the cleaved form of the spike at the size ~100 kDa was predominantly present [30]. With mutation at the S1/S2 cleavage site, the spike expressed in this system was detected at the size of a full-length spike, which coincides with other studies [35,48]. However, they all could form VLPs when visualized with TEM (Figure 2B). Our study suggested that only S, E, and M are required for SARS-CoV-2 VLP assembly. These results indicate that our SARS-CoV-2 VLPs only require S, E, and M to form VLPs. This is consistent with multiple studies that have demonstrated that the co-expression of E and M coronavirus proteins without nucleocapsid (N) led to the efficient release and assembly of enveloped coronavirus-like particles [44,49,50]. SARS-CoV-2 VLPs containing only the 2P mutation were selected for fed-batch analysis and scaling up.

It is crucial that protein expression from producer cells is maintained over a period of time. Consistent protein production ensures not only scalability in an industrial setting but also the reliability of product quality and homogeneity. Thus, the stable expression of the spike and membrane protein from the stable cell pool was investigated. Supernatants from the culture were collected when passaging the cells. The expression of spike and membrane proteins was maintained when the cell pool was cultured over six weeks (Figure 2C). The cell viability was more than 90% in every passage.

### 3.2. Feed Screening for SARS-CoV-2 VLPs Production in Fed-Batch Mode

**Figure 3:**
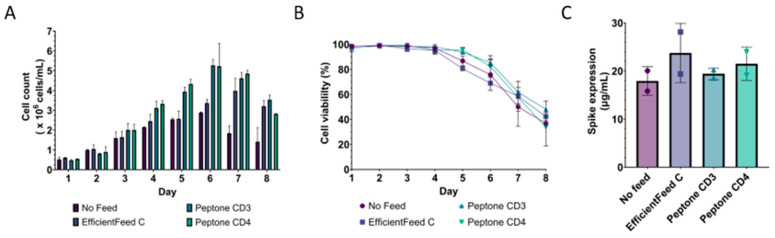
Expression of SARS-CoV-2 VLPs 2P in small-scale production. (**A**) Cell counts and (**B**) viability of the feed screening study in fed-batch mode from a shaken flask culture. (**C**) Spike expression level in the culture supernatant with varying types of feed on Day 7 quantitated with an immunoblot assay. The spike BA2.75 standard was used to construct a standard curve. Two biological replicates of the feed screening study were performed.

Three different feeds were evaluated in the shaken flask culture. Supernatants were harvested after seven days of culture. The spike expression level was quantitated using an immunoblot assay (Figure S1). All three feeds boosted spike level expression and cell growth compared to the unsupplemented culture (Figure 3A). Our study showed that the culture condition without feed had the lowest cell growth and viability. In addition, the fed-batch condition could prolong culture longevity in comparison to the unsupplemented batch culture. Peptone CD3 and peptone CD4 supplements provided higher cell growth than EfficientFeed™ C and the unsupplemented condition (Figure 3B). Nevertheless, the highest spike protein yield was obtained from EfficientFeed™ C, with peptone CD4 giving the second highest spike expression (Figure 3C). It is important to note that high cell counts can present challenges during harvesting and downstream processes.

### 3.3. Fed-Batch Production of SARS-CoV-2 VLPs in Scalable Stirred-Tank Bioreactor

**Figure 4:**
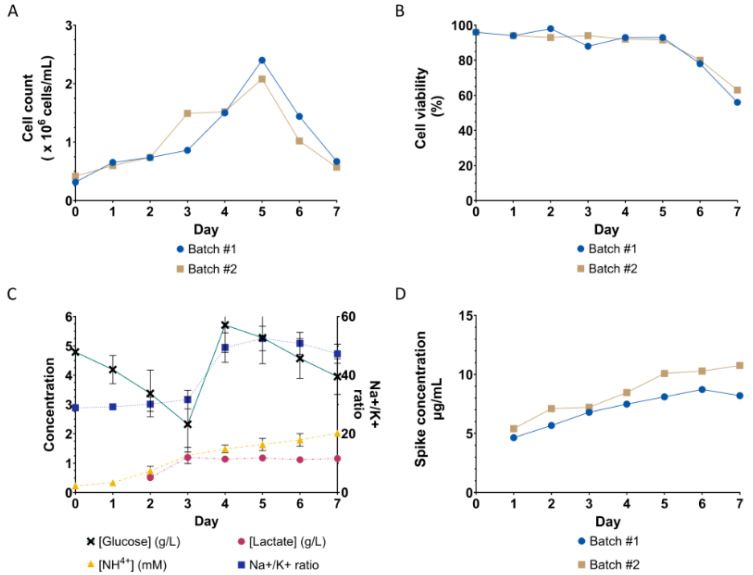
Performance of stable HEK293-F cell pool expressing SARS-CoV-2 VLPs in a 2 L fed-batch bioreactor with EfficientFeed™ C supplement. (**A**) Cell counts and (**B**) viability of the stable cell pool were monitored daily. The production lasted 7 days when the cell viability dropped below 70%. The maximum cell density was 2.3 × 10^6^ cells/mL on Day 5 and gradually decreased. (**C**) Metabolite analysis was performed daily on the collected culture supernatant. (**D**) Quantitation of spike expression in the collected culture supernatant every 24 h using immunoblot analysis (Figure S2).

The scaling-up feasibility of a stable HEK293-F cell pool to produce VLPs was evaluated in a stirred-tank bioreactor. The fed-batch condition was performed using EfficientFeed™ C as it provided the highest cell specific productivity (Figure 3). Scale-up bioreactor parameters for HEK cell culture were employed, including dissolved oxygen at 40%, P/V at 1.12 W/m^3^, and a maximum aeration rate at 0.13 VVM. Cell counts and viability were monitored daily (Figure 4A). The expression of the spike protein was detected as early as 24 h post-seeding. Nutrients and glucose were rapidly consumed on Days 1 and 2, while lactate and ammonia were accumulated from Day 2 until the harvest day (Figure 4B). The supplement was added on Day 3 as the glucose level dropped below 3 g/L. Fast consumption of glucose in the early days indicates cell growth. Although cell density declined after Day 4, glucose was still consumed but at a lower rate. Cells started clumping on Day 3. Another study also observed cell agglomeration when culturing HEK293-F cells in a stirred-tank bioreactor [48]. Cell viability sharply declined after Day 5 and dropped below 70% on the day of harvest. The clumping was prominent from Day 5 to the harvest day, where the cell density was less than 1 million cells/mL. However, spike productivity steadily increased and was maintained until the day of harvest (Figure 4C).

### 3.4. Characterization of SARS-CoV-2 VLPs Expressed from a Stable HEK293-F Cell Pool

**Figure 5:**
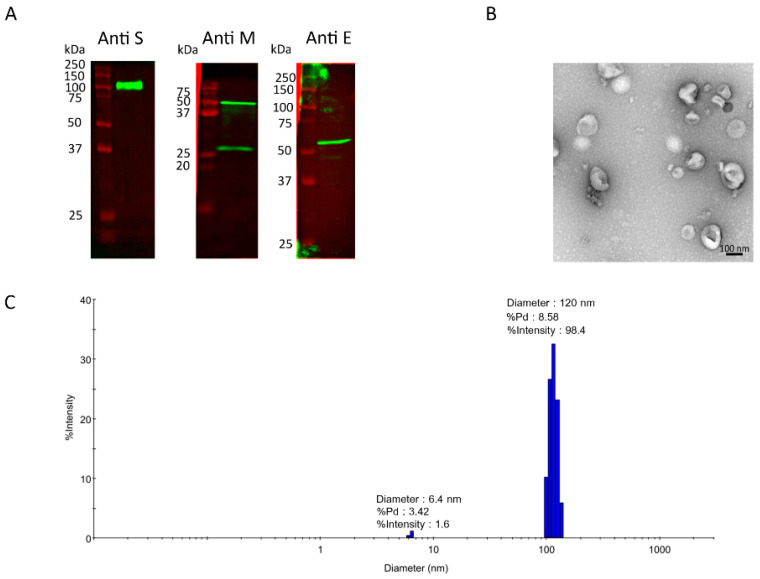
Characterization of SARS-CoV-2 VLPs produced from a stable HEK293-F cell pool cultured in a fed-batch stirred-tank bioreactor. (**A**) Structural proteins detection of CaptoCore 700 purified SARS-CoV-2 VLPs, including the spike, envelope, and membrane proteins. Spike detection indicates its decoration on the VLPs. (**B**) TEM image of purified SARS-CoV-2 VLPs. (**C**) Size distribution of purified SARS-CoV-2 VLPs measured with DLS.

To characterize the produced SARS-CoV-2 VLPs, the supernatant was harvested from a 2 L fed-batch bioreactor and chromatographically purified with a multimodal CaptoCore 700 column. Densitometric analysis of the SDS-PAGE image revealed that the purified SARS-CoV-2-containing sample comprised an estimated 9.4%, 19.6%, and 5.1% of total protein corresponding to the spike, envelope, and membrane proteins, respectively (Figure S3). The flowthrough containing purified VLPs from CaptoCore 700 was concentrated and buffer-exchanged. The concentrates were then analyzed using a Western blot with anti-S, anti-M, and anti-E antibodies. Figure 5A shows that the purified SARS-CoV-2 VLPs contained all three structural proteins. The productivity yield of purified SARS-CoV-2 VLPs is 176 µg per liter of culture, quantified using an immunoblot assay based on spike expression from two independent batches of culture.

TEM analysis was used to verify the integrity and particle assembly of the purified SARS-CoV-2 VLPs (Figure 5B). The observed particle diameter from TEM ranged from 80 to 120 nm. The hydrodynamic diameter measured with DLS was approximately 120 nm (Figure 5C). The sample was monodispersed, indicating homogeneity. These data are consistent with the authentic wild-type SARS-CoV-2 virions and SARS-CoV-2 VLPs expressed from mammalian cells [48,51,52].

### 3.5. Functionality of SARS-CoV-2 VLPs Expressed from a Stable HEK293-F Cell Pool in a 2 L Stirred-Tank Bioreactor

**Figure 6:**
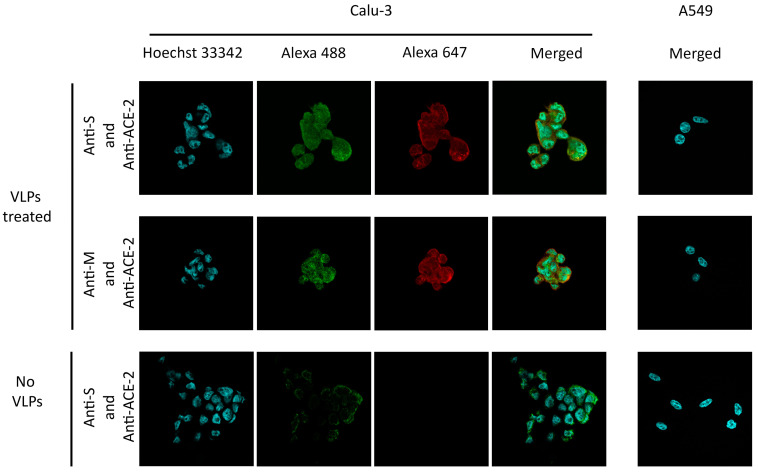
SARS-CoV-2 VLPs receptor binding assay. Calu-3 and A549 cells were incubated with VLPs and mock buffer (no VLPs). VLPs in the cells were visualized by detecting M (Alexa Fluor^®^ 647, red) and S (Alexa Fluor^®^ 647, red) in both cell lines with respective antibodies. The ACE-2 receptor was probed with an anti-ACE-2 antibody (Alexa Fluor^®^ 488, green). Nuclei were stained with Hoechst 33342 (blue). The A549 cell line did not express the ACE-2 receptor when the cell was probed with an anti-ACE-2 antibody.

To observe the functionality of our purified SARS-CoV-2 VLPs, the target receptor binding on the host cell was investigated. ACE-2 acts as a receptor for SARS-CoV-2 entry. Therefore, the Calu-3 lung adenocarcinoma cell line that naturally expresses the ACE-2 receptor served as a target host cell in this study. The A549 lung carcinoma cell line that does not express the ACE-2 receptor was employed to examine the VLPs’ uptake specificity. The cells were plated onto chamber slides and incubated with the purified VLPs or mock buffer. The expression of the ACE-2 receptor in both cell lines was verified with an anti-ACE-2 antibody. VLPs were detected with anti-M and anti-S antibodies only in Calu-3, ACE-2-expressing cells (Figure 6). As expected, VLPs were not detected in the A549 cell line, which does not express the ACE-2 receptor (Figure 6). Our results indicate that the produced SARS-CoV-2 VLPs entered the cells in a receptor-dependent manner. The specific susceptibility of the cell entry is similar to the authentic SARS-CoV-2 [53,54].

## 4. Discussion

This study demonstrated an economical and rapid method to generate a stable HEK293-F cell pool expressing SARS-CoV-2 VLPs. The stable cell pool was established with a simple and cost-effective transfection technique using the easily accessible reagent PEI. Small-scale protein production serves as a foundation for optimizing processes and can facilitate scaling up, which is crucial for industrial production settings. Our stable cell pool was able to produce SARS-CoV-2 VLPs in a stirred-tank bioreactor with a reproducible outcome. The purified SARS-CoV-2 VLPs were properly assembled and functional. TEM revealed fully intact spherical particles. Since the authentic virus utilizes ACE-2 to gain access to the cell for infection [55], our expressed particles could mimic the native virus as they specifically entered ACE-2-expressing cells when a traditional in vitro SARS-CoV-2 cell infection protocol was used [33]. In addition, a panel of spike gene variations was also designed, including unmodified BA 2.75, 2P mutation, and 2Pplus, having the 2P mutation and furin cleavage site mutation. The incorporation of the spike protein varied with different mutations. The spike of 2P yielded the highest spike incorporation of VLPs. The assembly of the particles was observed in all three spike designs, as demonstrated by TEM analysis. One potential area for further study could be the comparison of in vivo immunogenicity among various spike designs.

We have shown that expressing S, E, and M is sufficient for SARS-CoV-2 VLP assembly. Our vector designs can be quickly adaptable for new S gene designs if needed, such as in the case of new variants of concern. We co-transfected mammalian-based expression vectors, one bearing a gene for the envelope–membrane protein and the other encoding the spike protein. An expression vector that contained both structural proteins, M and E, was previously shown to improve the particle assembly efficiency of a coronavirus [56]. The requirement of N protein is debatable, as studies from Swann [43] and Naskalska [33] reported that N is indispensable for SARS-CoV-2 VLPs formation in opposition to Boson’s work [42]. Meanwhile, the recent effort from Hirschberg and the team developed an inducible stable mammalian cell line that expressed all four structural proteins for SARS-CoV-2 VLPs production in a bioreactor using a more complicated approach, non-viral transposon system with Expi293 cells [48].

Our stable cell pool exhibited protein expression stability, which is desirable for stable cell expression at a larger scale production with extended periods of cell expansion before production. In terms of production, various fed-batch strategies were implemented to boost expression. Here, our simple mammalian stable cell pool provided about 180 µg per liter of culture productivity in a 2 L bioreactor of purified SARS-CoV-2 VLPs based on spike expression. The yield and stability of VLPs may be further improved by incorporating the nucleocapsid protein into the particle [45]. However, HEK293-F cells tend to aggregate, which can become challenging for large-scale production [57,58]. Multiple approaches can be utilized to improve cell aggregation, such as the control of Ca^2+^ and Mg^2+^ content in the culture, sheer stress, the stirring speed, and the supplementation of an anti-clumping agent [58,59,60].

The conventional method for purifying VLPs using ultracentrifugation has been a limitation in developing a large-scale VLPs production process. To overcome this challenge, scalable downstream processes are required. We demonstrated that, with a simple tag-free single multimodal chromatography, the particles produced from a stirred-tank bioreactor could be purified with an efficiently scalable procedure. However, a multi-column design may help to improve the purity profile, such as incorporating a weak anion exchange resin as a capture step in a neutral pH buffer [61]. Affinity mode can also be featured since the spike protein is heavily glycosylated. It has been observed that lentil lectin resins are effective in purifying the spike protein [32]. Although this study demonstrated scalability up to a 2 L bioreactor, transitioning from laboratory-scale bioreactors to larger industrial-scale vessels to produce material for preclinical toxicology study can be readily achievable with a scale-up approach with parameters such as power/volume, mass transfer coefficient, and tip speed [2,62]. Given our current production performance, a 50 L scale production would be sufficient for comprehensive toxicological evaluations and protective immunization studies [63,64].

## 5. Conclusions

In summary, we have demonstrated an expedited development timeline of a scalable SARS-CoV-2 VLPs production platform with a robust outcome by using an economical approach to establish a stable HEK293-F cell pool. The non-clonal mammalian-cell-based approach has been a highly attractive alternative to typical cell line development in the production of biotherapeutics. In particular, this strategy potentially minimizes upfront costs and accelerates the production time of novel biotherapeutics for early development phases.

## Data Availability

All related data and methods are presented in this paper. Additional inquiries should be addressed to the corresponding authors.

## Supplementary Materials

The following supporting information can be downloaded at: https://www.mdpi.com/article/10.3390/vaccines12060561/s1, Figure S1: Immunoblot assay for spike quantitation from feed screening study in shaken flask culture. Figure S2: Immunoblot assay for spike quantitation from 2 L fed-batch stirred-tank bioreactor. Figure S3: Purification of SARS-CoV-2 VLPs from CaptoCore 700. (A) SDS-PAGE analysis and (B) Western blot analysis, probed with anti-SARS-CoV-2 S, of each fraction from 97 mL column CaptoCore 700 purification. Lane 1: Preload clarified supernatant, Lane 2: Flowthrough of the supernatant, Lane 3: The flowthrough sample after 30x concentration and buffer-exchange with 100 kDa Amicon and filtration with a 0.2 µm filter.
