# Supplementary material for: Rapid and Scalable Production of Functional SARS-CoV-2 Virus-like Particles (VLPs) by a Stable HEK293 Cell Pool
Source: Vaccines (Basel). 2024 May 21;12(6):561. doi: 10.3390/vaccines12060561 (PMC11209123; doi:10.3390/vaccines12060561)
Supplement: Supplementary file 1 [file vaccines-12-00561-s001.zip › vaccines-2966369-supplementary.pdf]

Supplementary Materials

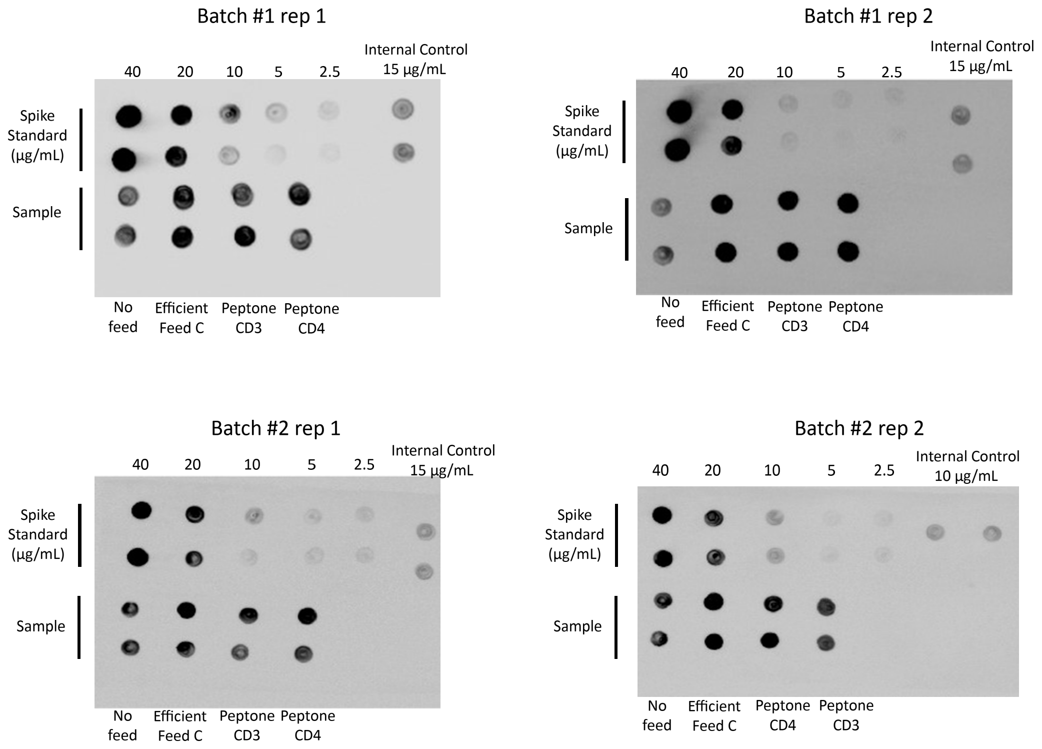

Figure S1. Immunoblot assay for spike quantitation from feed screening study in shake flask culture.

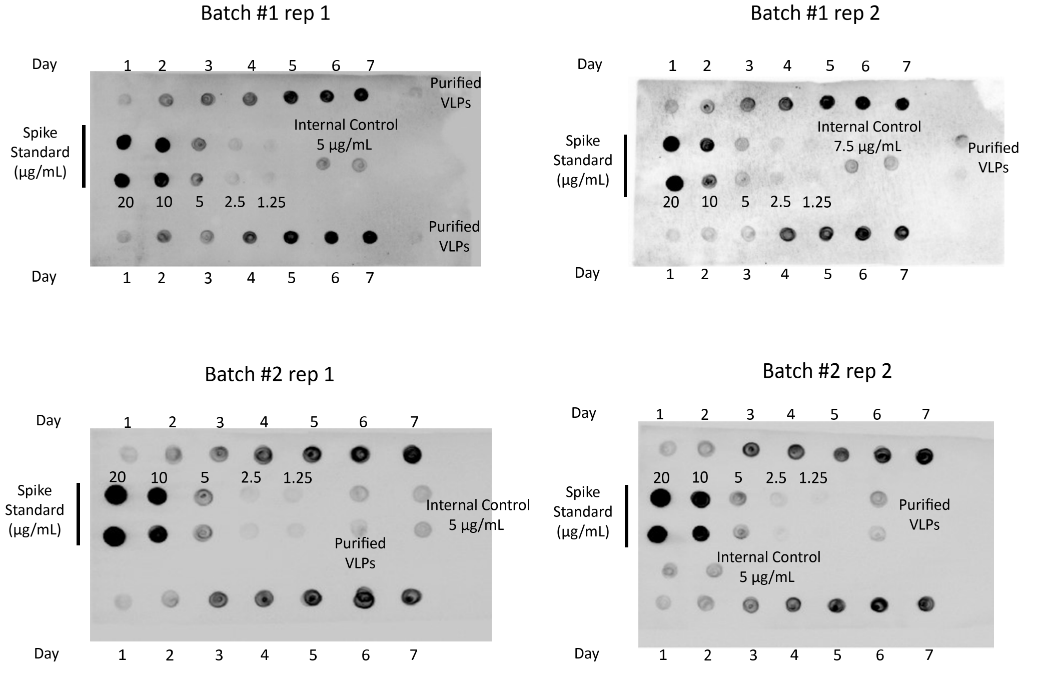

Figure S2. Immunoblot assay for spike quantitation from 2-L fed-batch stirred-tank bioreactor.

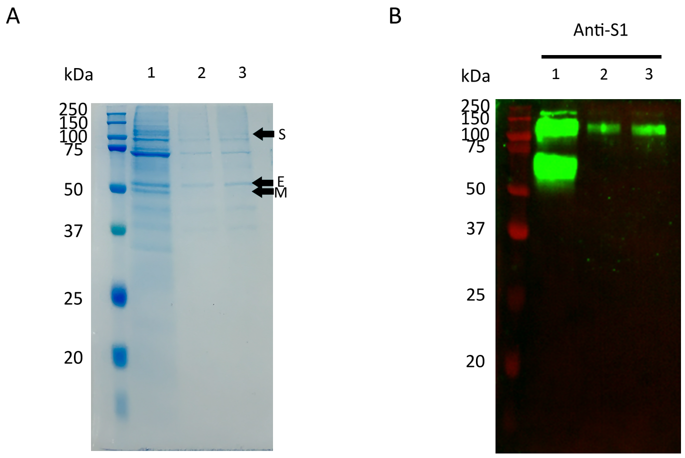

Figure S3. Purification of SARS-CoV 2 VLPs from CaptoCore 700. (**A**) SDS-PAGE analysis and (**B**) Western blot analysis, probed with anti-S1, of each fraction from 97-mL column CaptoCore 700 purification. Lane 1: Preload clarified supernatant, Lane 2: Flowthrough of the supernatant, Lane 3: The flowthrough sample after 30x concentration and buffer-exchange with 100 kDa Amicon and filtration with 0.2 μm filter.
